# Massive subchorionic hematoma (Breus’ mole) presents a variety of ultrasonic appearances: A case report and literature review

**DOI:** 10.1002/ccr3.2036

**Published:** 2019-03-05

**Authors:** Fumi Yanagisawa, Shigeru Aoki, Mizuha Odagami, Etsuko Miyagi

**Affiliations:** ^1^ Perinatal Center for Maternity and Neonatal Yokohama City University Medical Center Yokohama Japan; ^2^ Department of Obstetrics and Gynecology Yokohama City University Hospital Yokohama Japan

**Keywords:** Breus’ mole, intraplacental fluid‐fluid level, massive subchorionic hematoma, placentomegaly

## Abstract

Massive subchorionic hematoma (MSH) presents a variety of ultrasonic appearances. Placentomegaly with fetal growth restriction should be included as one of the differential diagnoses for intraplacental MSH. Care management of MSH requires to be tailored to each individual's responses while taking the NICU's capabilities and the patient's wishes into consideration.

## 1 INTRODUCTION

Massive subchorionic hematoma (MSH) is a large maternal blood clot with a thickness of at least 1 cm, that separates the chorionic plate from the villous chorion.^1^ Especially when presenting preplacentally, MSH is commonly termed “Breus’ Mole” and has been linked with poor pregnancy outcomes such as fetal growth restriction (FGR) and intrauterine fetal death (IUFD).^2^, ^3^, ^4^ The estimated incidence of this condition is low (1/3000 pregnancies).^3^ Almost all studies of this condition were reported as case reports or case series, and it is one reason why its pathophysiology is still obscure. Although ultrasound examination and magnetic resonance imaging (MRI) are useful for diagnosis, determining a diagnosis is not always straightforward.^3^, ^5^, ^6^, ^7^, ^8^ Here, we report a case of placentomegaly with FGR caused by MSH and review the available literature on MSH.

## 2 CASE PRESENTATION

**Figure 1:**
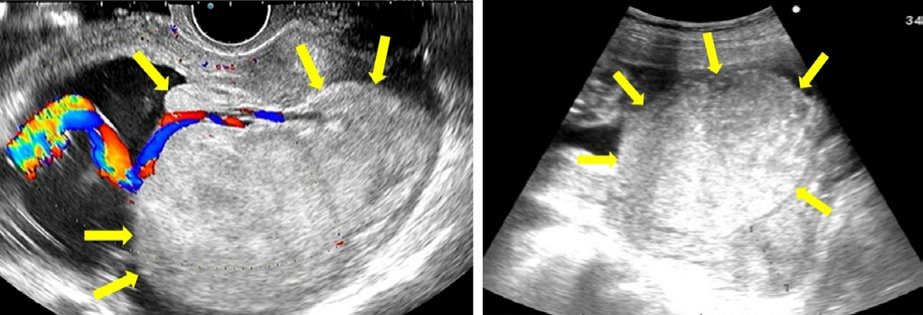
Ultrasonographic image shows (arrows) a spherically enlarged placenta; color Doppler sonography shows the umbilical cord

**Figure 2:**
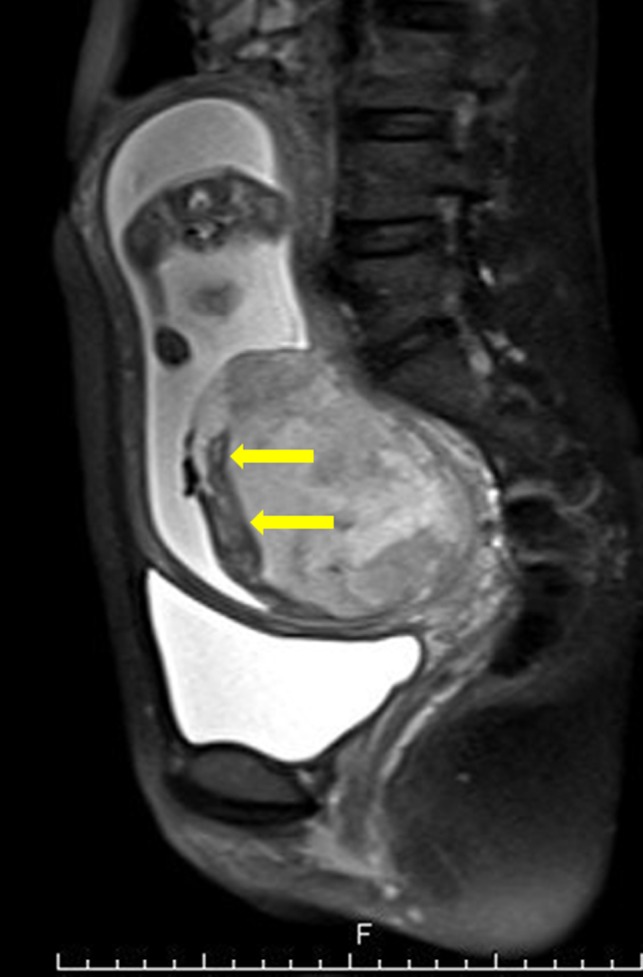
MRI T2‐weighted images at gestation of 24 wk and 3 d. MRI shows that the placenta is heterogeneous and enlarged. Arrows indicate fluid‐fluid level in the image. Placenta accreta was not observed

**Figure 3:**
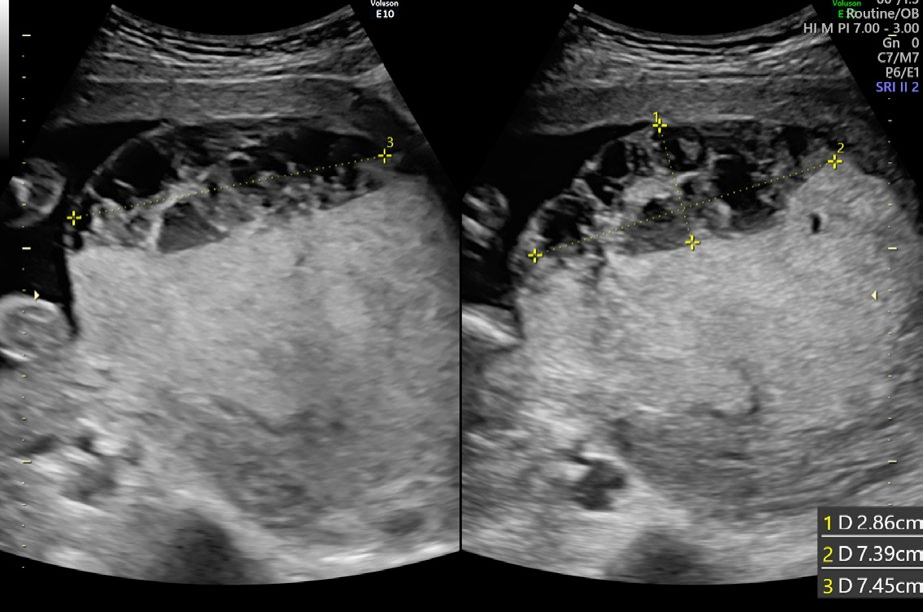
Ultrasonographic image shows intraplacental fluid‐fluid level (24 wk 4 d of gestation). Color Doppler sonography showed no blood flow in the reticulate sonolucent areas

A 40‐year‐old woman (gravida, 2; parity, 1) was referred to our hospital at 23 weeks and 2 days of gestation due to placenta previa and severe FGR. Her previous delivery was a normal vaginal delivery with manual removal of the placenta. An ultrasound examination showed no fetal anomaly, however, the estimated fetal body weight (EFBW) was 258 g (−3.7 SD), severe FGR was observed, and both placenta previa and enlargement of the placenta (8 × 9 × 7 cm) were confirmed. The umbilical cord was inserted into the center of the placenta, and the umbilical artery end‐diastolic flow velocity was normal (Figure 1). Blood sample results revealed the following: Hb, 12.4 g/dL (normal range: 11.6‐4.8 g/dL); platelet count, 99 000/μL (158 000‐348 000/µL); APTT, 27 seconds (24.5‐38.7 seconds); PT‐INR, 0.88 (0.90‐1.10); D‐dimer levels, 0.9 µg/L (≦1.0 µg/L). The thrombocytopenia was diagnosed as gestational thrombocytopenia and not idiopathic thrombocytopenic purpura (ITP) by the hematologist. The patient was not taking aspirin or any other anticoagulants and did not have thrombophilia, such as antiphospholipid syndrome (APS), protein S deficiency, or protein C deficiency. The condition was diagnosed as idiopathic placentomegaly with severe FGR. Pelvic MRI at 24 weeks and 3 days of gestation revealed that the placenta showed internal heterogeneity and enlargement. Furthermore, placenta previa and a fluid‐fluid level on the fetal side of the placenta with no blood flow were identified (Figure 2). Ultrasound examination at 24 weeks and 4 days of gestation confirmed the presence of an MSH in a clear fluid‐fluid level forming on the side of the fetus in the placenta, 2.8 × 7.4 cm in size, with no blood flow by color Doppler sonography (Figure 3). At 25 weeks and 3 days of gestation, the EFBW was 410 g. Color Doppler sonography showed absent end‐diastolic flow velocity and indicated that termination of the pregnancy would soon be required; therefore, betamethasone was administered to accelerate fetal lung maturation. At 26 weeks and 6 days of gestation, strong uterine contractions and genital bleeding led to suspicion of placental abruption, and thus, an emergency cesarean delivery was performed. The placenta was 12.0 × 9.0 × 1.5 cm in size and weighed 153 g, and a hematoma of 8.0 × 6.0 cm in size was macroscopically identified in the subchorionic region and confirmed by histological examination. The maternal side of the placenta was normal. MSH was confirmed by postpartum histological findings. A male infant weighing 486 g (−3.3 SD) was delivered with Apgar scores of 2 and 5 at 1 and 5 minutes, respectively. He was admitted to the neonatal intensive care unit (NICU). He died nine days postpartum due to respiratory and heart failure. The mother's postoperative course was uneventful, and she was discharged in good health on the 6th postpartum day.

## 3 DISCUSSION

This case highlights two points: Intraplacental MSH should be considered as a cause of placentomegaly, and that MSH ultrasound findings are highly variable.

First, physicians should consider intraplacental MSH as a differential diagnosis when placentomegaly with FGR is observed. Placental thickness typically does not exceed 40 mm.^9^ In this case, the placenta had swollen to 8 × 9 × 7 cm in size. Although placentomegaly often results from villous enlargement, there are several causes of placentomegaly such as hydrops, infections caused by syphilis, toxoplasmosis, cytomegalovirus or parvovirus, and placental mesenchymal dysplasia (PMD).^10^ Rarely, as in this case, a placental hematoma may be one such cause of placentomegaly. When an unknown placentomegaly, especially FGR, is present, an intraplacental MSH should be considered as a differential diagnosis.

Secondly, the ultrasound findings of MSH are highly variable, and the pregnancy outcome is poor. Table 1 shows the results of a literature review performed by searching MEDLINE for “massive subchorionic hematoma” and/or “Breus’ mole” published in English after 2000.^2^, ^3^, ^4^, ^5^ There were 43 cases, including the case outlined here. The average gestational age at delivery was 29.9 weeks, birth weight was 1076 g, mortality rate was 46.5% (20/43), term delivery was only 9.3% (4/43), and FGR occurred in 55.8% (24/43) of these cases. Perinatal outcomes were markedly poor. FGR occurs due to utero‐placental insufficiency, and when a hematoma occurs near the umbilical cord attachment, the umbilical vessels can be compressed; it has been suggested that perinatal outcomes in these cases are considerably worse.^23^ Alanjari et al^2^ reported that the perinatal outcome depends on gestational age at delivery and the degree of FGR, and that normal umbilical artery Doppler at presentation is a favorable prognostic sign. A total of 90.7% (39/43) of cases were identified antenatally. However, these data are biased given that there are many unreported or unrecognized cases of MSH. Furthermore, the mortality rate reported here is based on a review of the available literature review and, as such, there may be a selection bias. Ultrasonic findings of subchorionic hematoma are highly varied, and the condition is not always easy to distinguish from placental parenchyma. Himoto et al^5^ reported that MRI for MSH is useful to confirm a diagnosis and follow‐up when used in combination with ultrasonography. In this case, an MSH diagnosis was made through ultrasonic imaging of the fluid‐fluid level with no blood flow, using color Doppler sonography performed at 24 weeks and 4 days of gestation in addition to MRI. Nishijima et al^4^ reported that the fluid‐fluid level occurs in the early stage of the hematoma. Wang et al^22^ reported a case where fluid‐fluid levels continued from week 23 to week 29 of gestation, and that fluid‐fluid levels are a subacute stage of hemorrhage rather than an early stage of hematoma. In this case, the fluid‐fluid level temporarily appeared at 24‐week gestation. This indicates that the fluid‐fluid levels are caused by a sedimentation effect due to sequential bleeding, with parts of the clot being solid and others being liquid; taken together, these findings suggest that there is a possibility of this occurring at any time.

**Table 1:** Cases of massive subchorionic hematoma

	Reference	Case	Age	Gravida parity	GA at delivery (wk)	Birth weight (g)	FGR	Neonatal course	GA at US identification (wk)
1	Kojima et al (2001)^8^	1	25	G1P0	32	490	Yes	IUFD	28
2	Nishida et al (2001)^11^	1	28	G1P0	33	‐	Yes	Lived	22
3	Usta et al (2004)^12^	2	21.5	Nullipara 1 multipara 1	36.5	2885	None	2 lived	20.5
4	Fisteag‐Kiprono et al (2005)^7^	1	35	G1P0	23	272	Yes	IUFD	19
5	Nishijima et al (2005)^4^	1	29	G1P0	27	376	Yes	IUFD	25
6	Koçak et al (2006)^13^	1	22	G1P0	28	1400	No	Died‐6 d (respiratory distress)	27
7	Matsudera et al (2006)^14^	1	26	G1P0	25	240	Yes	IUFD	21
8	Lee et al (2006)^15^	1	‐	G2P1	34	2200	No	Lived‐respiratory depression	18
9	Loi et al (2006)^16^	1	26	G1P0	32	1730	No	Lived‐Apgar 4/8	25
10	Madu et al (2006)^6^	1	37	G5P0	31	1470	No	Lived‐Apger 8/10	After birth
11	Gupta et al (2007)^17^	1	19	G1P0	32	1100	Yes	Lived	31
12	Fung et al (2010)^3^	10	29.2	Nullipara 8 multipara 2	29.8	1305	No 8	6 lived	21
							Yes 2	4 died	
13	Asada et al (2011)^18^	1	‐	G1P0	27	471	Yes	Lived‐Apgar 4/8	25
14	Giri et al (2011)^19^	1	18	G1P0	25	268	Yes	IUFD	20
15	Yamada et al (2012)^20^	1	30	G2P0	31	789	Yes	Died‐2 h	‐
16	Himoto et al (2012)^5^	1	Early thirties	G4P2	34	1662	Yes	Lived‐Apgar 5/8	26
17	Alanjari et al (2013)^2^	14	‐	Nullipara 9 multipara 5	28.6	1029	No 5	7 lived	24.1
							Yes 9	7 died	
18	El‐agwany (2017)^21^	1	30	G1P0	34	‐	Yes	IUFD	28
19	Wang et al (2018)^22^	1	30	G2P0	29	1200	Yes	Lived‐Apgar 10	23
20	Current report	1	40	G2P1	26	486	Yes	Died‐9 d	24
	Total	43	27.9	Nullipara 32 multipara 11	29.9	1076.3	24/43	Lived 23/43	23.8

The data are represented as means.

In conclusion, placentomegaly, particularly when accompanied by FGR, should be included as one of the differential diagnoses for intraplacental MSH. Although it is unknown if an antenatal diagnosis of MSH improves the perinatal outcome, the emergence of MSH complications should not be a reason to discourage a cesarean section.^18^ Care management, including termination, requires to be tailored to each individual's responses while taking the NICU's capabilities, the patient's wishes, and other relevant factors into consideration.

## CONFLICT OF INTEREST

None declared.

## AUTHOR CONTRIBUTIONS

FY, SA: contributed to the study design and finalization of the manuscript. MO: wrote the first draft of the manuscript. EM: provided the study design and supervised the study.
